# FAAH inhibitors in the limelight, but regrettably 

**DOI:** 10.5414/CP202687

**Published:** 2016-05-18

**Authors:** Christophe Mallet, Claude Dubray, Christian Dualé

**Affiliations:** 1 Inserm U1107 «Neuro-Dol»,; 2 Univ-Clermont1, and; 3 CHU Clermont-Ferrand, Centre de Pharmacologie Clinique (Inserm CIC1405), Clermont-Ferrand, France

**Keywords:** fatty acid amide hydroxylase, endocannabinoids, phase I, serious adverse event, brain injury

## Abstract

Abstract. This short review focuses on the recent drug development of FAAH inhibitors, as recent serious adverse events have been reported in a phase I study with a compound of this class. The authors overview the potential interest in targeting FAAH inhibition, the current programs, and the available information on the recent dramatic events.

## Guest Editorial 

In January 2016, we were informed of the exceptional occurrence of serious adverse events (SAEs) in a phase I clinical trial conducted by the Biotrial Pharmacology Center (Rennes, France) on behalf of Bial-Portela & Ca. SA (São Mamede do Coronado, Portugal). The trial involved the compound BIA 10-2474, a drug designed to inhibit fatty acid amide hydrolase (FAAH). After two initial phases (single escalating doses up to 100 mg, and kinetics-food interaction studies) without any untoward SAE, the phase in question, which intended to examine the effect of multiple doses (5 or 6 daily doses), resulted in SAEs in 6 participants, who had all been administered the highest tested dose (50 mg). This was a threshold effect, since no SAE had been reported with the lower dose of 20 mg given to the volunteers previously. The most serious symptoms had central neurological features, the worst being those associated with a single case of coma which rapidly lead to brain death. Of the other 5 hospitalized participants, 2 had serious neurological damage (with clinical improvement apparently occurring within a few days). Because of these events, the trial was immediately suspended. Further information (including the protocol of the trial) is available on the website of the French National Agency for Medicine and Health Product Safety (ANSM)^1^. The agency has also recently published the summarized conclusions of a temporary specialized scientific committee^2^. Although no definitive conclusion can be drawn at the present time regarding the causes, the seriousness of the events has undoubtedly and, in this case regrettably, brought the development of FAAH inhibitors/inactivators into the limelight. 

FAAH inhibitors/inactivators have been developed because of their ability to increase the concentration of endocannabinoids. Endocannabinoids are lipid mediators released on demand from membrane phospholipid precursors. Their targets are the cannabinoid receptors CB_1_ and CB_2_, but other receptors can be involved in their action, such as GPR_55_, peroxisome proliferator-activated receptors (PPARs) and vanilloid receptors (TRPV_1_). This system has been implicated in a wide range of physiological processes such as those associated with chronic pain, metabolic disorders, psychoses, nausea and vomiting, depression, and anxiety disorders (see [1, 2, 3, 4, 5] for reviews). Some exogenous cannabinoids acting on CB_1–2_ are currently used in therapeutics (e.g., Bedrocan^®^, Bedrobinol^®^, Bediol^®^, Bedica^®^, Cesamet^®^, Marinol^®^, Sativex^®^) involving a variety of indications such as anorexia, neuropathic pain and multiple sclerosis, depending on the country in which the drugs are marketed. However, such treatments may have neurological side effects (including impairment of cognition and motor functions and a predisposition to psychoses), notably when these agents are used for long-term treatment [6, 7]. 

Increasing the concentration of endocannabinoids, rather than administering exogenous agonistic agents, would reduce cannabinoid-like adverse events. This strategy can be achieved through the inhibition of catabolic enzymes, notably FAAH, an integral membrane enzyme that hydrolyzes the fatty amide family of lipid transmitters including the most thoroughly studied endocannabinoid, N-arachidonoylethanolamide (anandamide) [8]. FAAH also degrades several related fatty acid amides which have diverse biological functions and mechanisms of action [9]. FAAH-deficient mice have enhanced levels of anandamide and display a CB_1_ receptor-mediated hypoalgesic phenotype [10, 11]. Pharmacological inhibition of FAAH increases fatty acid amide concentrations in both rats and humans [12, 13]. This strategy seems to be successful in animal models of anxiety and depression [3], sleep disorders [14], and nociceptive or neuropathic pain [12, 15, 16]. Interest in this pharmacological pathway is illustrated by the numerous molecules under development. Among the most advanced programs are those concerning the compounds PF-04457845, JNJ-42165279, SSR-411298, V-158866, and URB597^3^. The disorders for which these agents are being tested are mostly neuropsychiatric, such as pain conditions, depression, anxiety disorders, and phobias, Tourette syndrome, and symptoms associated with cannabis withdrawal. The available data from completed clinical trials indicate that FAAH inhibitors are well tolerated. A phase I study of PF-04457845 (developed by Pfizer, New York, NY, USA) showed that, compared to placebo, the increase in somnolence was only mild, and that there were no effects on cognitive function [13]. The corresponding phase II study demonstrated that this agent had a safety profile indistinguishable from placebo, where the main treatment-related side effect was dizziness [17]. A phase I study of JNJ-42165279 (developed by Johnson & Johnson Pharmaceutical, New York, NY, USA) found few side effects and all were of mild intensity. A slight and transient increase in liver transaminases was observed at the highest doses in a few cases [18]. In a phase II study with SSR-411298 (Sanofi, Gentilly, France) in patients with depression, the rate of adverse events was similar in the treatment and the placebo group. Headache, suicidal ideation, diarrhea, dizziness, and nausea were the most frequent adverse effects observed in the treatment group [19]. A major observation was that FAAH inhibitors do not apparently induce those adverse effects commonly associated with exogenous cannabinoids, such as impairment in cognition, motor coordination, and psychoses. However, it must be noted that the effects of chronic treatment are still largely unknown. It is also of interest that in two randomized, double-blind, placebo- and active drug-controlled clinical trials, no clinical efficacy of the tested FAAH inhibitor could be demonstrated. This was the case with PF-04457845 on pain due to osteoarthritis [17], and with SSR-411298 in elderly patients with major depressive disorders [19]. Following announcement of the accident at Biotrial, the two JNJ-42165279 trials were also suspended as a precautionary measure. 

Since these events came to light, many researchers and clinicians have questioned whether the type of adverse events observed are directly related to an inhibition of FAAH, and speculate that they may result from an interaction with other pharmacological targets, specific to BIA 10-2474 and which only become important above a certain concentration threshold. An answer to this question is highly important since on the one hand, this type of adverse reaction could potentially occur with all substances belonging to this class of drugs but on the other, this would not apply if a different target, specific to this particular drug was involved. The putative role of a metabolite has also been mentioned by the experts commissioned by the ANSM. They have since rejected the hypothesis of a manufacturing defect, and state that the preclinical studies – including that in which dogs died – had met the prerequisites for a first-in-man study. 

The protocol document of the above mentioned phase I study of BIA 10-2474 shows that the study design is conventional and was apparently strictly adhered to. However, more precise information concerning the extent of the brain damage in the healthy study volunteers who suffered neurological symptoms in the days following the first 5 or 6 BIA 10-2474 administrations is still awaited. The short report which has so far been published by ANSM mentions symptoms related strictly to cerebral functions, with “very fast appearance”, with homogeneous aspects but where there are between-subject differences in severity and progression. MRI observations showed the presence of “micro-cerebral tissue damage of varying severity and with unusual topography”. On the other hand, this report has not provided a credible lead concerning possible mechanisms involved. 

The fact that the adverse events were observed in 5 out of the 6 subjects who received 50 mg per day of BIA 10-2474 excludes the possibility of an idiosyncratic reaction in an individual with a particular phenotype. Thus, a concentration-dependent effect, with some between-subject variability, seems more likely. In animals, the pharmacokinetic half-life is relatively long and the fact that the accident happened after several administrations, argues for a product accumulation in which toxic levels are reached within a few days. This may result from low clearance rates and elimination pathways in humans that differ from those in the experimental animals used during product development. Although this hypothesis cannot be excluded, it does not appear to be a likely explanation because BIA 10-2474 does not interact directly with receptors, but rather inhibits an enzyme involved in the catabolism of endocannabinoids. In biological systems, inhibition of one metabolic pathway is often compensated by activation of alternative pathways in order to restore or at least partially restore the substrate balance and status quo. In support of this viewpoint is the fact that phenomena resembling those seen in humans with BIA 10-2474 have not been reported in the literature in any of the numerous animal studies with various inhibitors of FAAH, even when high doses have been used or, as in the case of rodents, when endocannabinoids have been administered by intracerebral injection. That FAAH-deficient mice (knock-out) thrive and exhibit no abnormal behavioral symptoms supports the hypothesis even though not yet conclusive, that BIA 10-2474 itself or a metabolite thereof and not the inhibition of FAAH is the cause of the adverse reactions. The analysis of BIA 10-2474 plasma concentrations in the healthy subjects who were included in the repeated-dose study should provide information important for answering two fundamental questions, namely: (i) Does drug or metabolite accumulation occurring in humans result from low clearance or the presence of saturable metabolic pathways? (ii) Is there a relationship between the magnitude of blood concentrations and the severity of neurological symptoms in subjects who received the highest dose of 50 mg? In the course of the coming weeks or months it is hoped that information will come to light enabling a distinction to be made between the possible target specificities of BIA 10-2474 and those of other FAAH inhibitors. This information is vitally important since the fate of this class of drug and the marketing potential of FAAH-inhibitors depend on it. 

## Conflict of interest 

The authors have no conflict of interests to declare. 

## Footnotes

^1^http://ansm.sante.fr/S-informer/Actualite/Essai-clinique-BIA-102474-101-Bial-Biotrial-Chronologie

^2^http://ansm.sante.fr/Dossiers/Essai-Clinique-Bial-Biotrial/Essai-clinique-BIA-102474-101-du-laboratoire-BIAL/(offset)/0

^3^See refs. on Clinicaltrials.gov: NCT02134080, NCT01618656, NCT00981357, NCT02498392, NCT02432703, NCT00822744, NCT01439919, NCT01748695 & NCT00916201.
